# Commentary: Episodic Memory Retrieval Functionally Relies on Very Rapid Reactivation of Sensory Information

**DOI:** 10.3389/fnhum.2016.00196

**Published:** 2016-04-29

**Authors:** Holly J. Bowen, Sarah M. Kark

**Affiliations:** Department of Psychology, Boston CollegeChestnut Hill, MA, USA

**Keywords:** reactivation, retrieval, episodic memory, ecphory, oscillations, TMS, visual cortex

Several prominent memory theories are predicated on the idea that retrieval relies on reactivation of processes engaged during encoding, a process known as “ecphory” (Tulving, 1983). Functional magnetic resonance imaging (fMRI) work has shown reactivation effects in visual cortex (e.g., Slotnick and Schacter, 2006; Kark and Kensinger, 2015) highlighting the importance of sensory reactivation to episodic memory. Such fMRI studies do not have the temporal resolution to distinguish ecphoric from retrieval processes, which are thought to be distinctly successive in time (Tulving, 1976). Studies of brain oscillations have provided insight into the temporal unfolding of memory. While many of these studies have focused on the memory-facilitating effects of *synchronization* in the γ/ θ bands, recent work indicates that *desynchronization* in α/β bands plays a complementary role in memory (Hanslmayr et al., 2012). Specifically, mathematical models of information theory suggest that while *synchronization* corresponds to cortical inhibition and facilitates information transfer (i.e., “fire together, wire together”), *desynchronization* in oscillatory activity corresponds to disinhibition and enhances the capacity for computational complexity, which facilitates an information-rich memory trace (Hanslmayr et al., 2016).

Oscillatory dynamics can be measured with electroencephalography (EEG) and manipulated using rhythmic transcranial magnetic stimulation (rTMS; Johnson et al., 2010), but until now have not been used in compliment to demonstrate a functional link between early oscillatory signatures of sensory reactivation and behavioral memory performance. Leveraging these techniques, Waldhauser et al. (2016) examined whether early sensory reactivation is necessary for episodic memory, in line with the theory of ecphory. In two experiments, participants encoded objects that were presented in the left or right visual field. During recognition, objects were presented centrally and participants made an old/new memory judgment followed by a spatial source judgment. Experiment 1 aimed to localize retrieval-related reactivation of oscillatory activity in sensory regions engaged during encoding, a potential neural marker of ecphory. EEG measured during encoding and retrieval revealed an early (~100–200 ms) signature of reactivation, as evidenced by desynchronization in the α/β band localized to the lateral occipital cortex in extrastriate cortex. In Experiment 2, rTMS during retrieval cue presentation disrupted retrieval-related desynchronization in lateral occipital cortex decreasing source memory for items originally presented to the contralateral—but not ipsilateral—visual field. These results provide the first evidence that early sensory reactivation is *causally* relevant for episodic memory and confirm that ecphory is critical for retrieval judgments.

To provide further insight into the basis of the link between sensory reactivation and ecphoric processes, there are a number of interesting directions for future research. First, evidence of reactivation in extrastriate cortex does not eliminate the possibility that reactivation processes might begin either earlier or later in the visual processing stream for some memory traces. Visual processing involves feedforward and feedback sweeps between earlier and later visual regions (Lamme and Roelfsema, 2000). While some studies report reactivation in early visual regions (Slotnick and Schacter, 2006), others posit an efficient retrieval process whereby reactivation occurs in higher-order portions of the ventral visual stream, without reactivation of early visual regions involved in processing of low-level visual features (Wheeler and Buckner, 2003). Second, as mentioned by the authors, ecphoric processes are not necessarily specific to the α/β band. Future work is needed to further probe the spatial (e.g., early vs. late visual cortex) and temporal (e.g., other frequency bands and onset of effects) characteristics—and the spatio-temporal interactions—that underlie ecphoric processes.

Further, questions remain regarding the underlying neuronal mechanisms that link α/β desynchronization in extrastriate cortex and memory performance. The authors find a significant—but small (7%)—decrease in memory performance for items presented to the contralateral visual field following rTMS. Thus, early reactivation might be necessary for *some* memories but clearly not *all* memories. A combined EEG-TMS approach is needed to confirm that high-frequency rTMS indeed *disrupts* desynchronization in extrastriate cortex. Conversely, additional evidence of causality could be revealed if rTMS can be used to induce extrastriate cortex into a *greater* state of α/β desynchronization, presumably driving a behavioral memory *enhancement*.

Beyond clarification of neuronal mechanisms, the findings of Waldhauser et al. afford a number of future directions to strengthen the argument that extrastriate reactivation is functionally relevant to episodic memory. Extrastriate cortex has distinct upper visual field and lower-visual field representations that map onto more ventral and dorsal portions, respectively (Strother et al., 2010). Presenting objects in all four visual quadrants and applying rTMS to more dorsal or more ventral extrastriate cortex could demonstrate quadrant-specific memory disruption. Such a paradigm would reduce the possibility that a participant guesses the correct source.

Second, Waldhauser et al.'s argument that extrastriate cortex reactivation is functionally necessary for *recollective* episodic retrieval could be strengthened with the addition of a remember/know judgment to separate memories that are recollected (i.e., include episodic content) from those that are familiar (i.e., devoid of contextual information). Recollection and familiarity have unique neural correlates (Yonelinas et al., 2002) and oscillatory signatures (Burgess and Ali, 2002), suggesting they are qualitatively different. If evidence for reactivation in the extrastriate cortex consistently occurs for items endorsed with “remember,” this would bolster their conclusions regarding ecphoric processes.

Finally, the question remains whether these findings are indicative of a general episodic memory mechanism. Although sensory reactivation appears functionally relevant visual-spatial source memory, it is not necessarily functionally relevant to episodic memory more generally, as they conclude. Episodic memory involves the conscious recollection of contextual information, but differing perspectives on how to test memory for context has led to the creation of a wide variety of episodic memory tasks. Results of different episodic memory paradigms are not always correlated, indicating the psychological and potentially neural processes that support these tasks are not always the same (Cheke and Clayton, 2013). Before concluding that early sensory reactivation supports episodic memory it is necessary to test the functional relevance of sensory reactivation to ecphoric processes using other episodic memory tasks.

## Author contributions

All authors listed, have made substantial, direct and intellectual contribution to the work, and approved it for publication.

## Funding

HB is supported by the National Institute of Mental Health (NIMH grant number R01MH080833 awarded to Elizabeth Kensinger). SK is supported by the National Science Foundation Graduate Research Fellowship Program (NSFGRFP grant number DGE1258923).

### Conflict of interest statement

The authors declare that the research was conducted in the absence of any commercial or financial relationships that could be construed as a potential conflict of interest.
